# Natural Compounds as Epimodulators in Epithelial Ovarian Cancer

**DOI:** 10.3390/epigenomes10020023

**Published:** 2026-04-01

**Authors:** Mélida del Rosario Lizarazo-Taborda, Julio César Villegas-Pineda, Holver Parada, Fabian Galvis, Javier Soto

**Affiliations:** 1 Laboratorio de Investigación en Cáncer e Infecciones (LICI), Departamento de Microbiología y Patología, CUCS, UDG, Guadalajara 44340, Jalisco, Mexico; melida.lizarazo1075@alumnos.udg.mx (M.d.R.L.-T.); julio.villegas@academicos.udg.mx (J.C.V.-P.); 2 Doctorado en Microbiología Médica, Departamento de Microbiología y Patología, Centro Universitario de Ciencias de la Salud, Universidad de Guadalajara, Guadalajara 44340, Jalisco, Mexico; 3 Facultad de Ciencias Médicas y de la Salud, Instituto de Investigación Masira, Universidad de Santander, Cúcuta 540003, Norte de Santander, Colombia; hol.parada@mail.udes.edu.co; 4 Grupo de Investigación Majumba, Facultad de Ciencias Básicas, Universidad Francisco de Paula Santander, Cúcuta 540003, Norte de Santander, Colombia; nestorfabiangs@ufps.edu.co

**Keywords:** phytochemicals, epithelial ovarian cancer, epigenetic changes

## Abstract

Epithelial ovarian carcinoma (EOC) is the most common type of ovarian cancer and represents the most lethal gynecologic neoplasm. EOC is usually diagnosed at late stages due to its nonspecific signs and symptoms. Although significant clinical advances have been made in other types of malignancies, EOC remains a disease that requires further biological research to identify new therapeutic targets or new treatment alternatives, as conventional approaches are often ineffective or lead to the development of resistance and unwanted side effects. There are a significant number of natural products from which commercially available drugs have been derived, largely for the treatment of cancer, but none of them focus on epigenetic changes in specific targets in EOC. Based on the above, this work focuses on describing the in vitro and in vivo findings from the last twelve years derived from the action of important phytochemicals on epigenetic targets in ovarian cancer, among other mechanisms of action, revealing that there is a significant gap to be bridged in terms of the transition from basic to applied research regarding the potential of plant-derived molecules as possible epidrugs in EOC.

## 1. Introduction

Epithelial ovarian carcinoma (EOC) is the most common type of ovarian cancer and represents the most lethal gynecological neoplasia. EOC is usually diagnosed in late stages due to its nonspecific signs and symptoms. In the latest GLOBOCAN 2022 report, 324,603 new cases and 206,956 deaths from EOC were recorded worldwide, confirming its high lethality rate [1]. EOC is a heterogeneous disease that is subdivided into five main types, and certain subtypes have other characterizations. For example, in HGSC (High-Grade Serous Carcinoma), four methylation profiles have been identified that encompass 168 differentially methylated genes in their promoter regions, three profiles related to microRNA expression, and four transcriptional subtypes called immunoreactive, differentiated, mesenchymal, and proliferative [2], which reflects the complexity and heterogeneity of this disease.

Initial response rates to platinum-based chemotherapy in EOC are high; however, disease recurrence occurs in almost 25% of early-stage cases (which are few) and in more than 80% of advanced cases [3], eventually developing resistance to platinum treatment. This resistance to conventional treatment remains one of the main obstacles to overcome in cancer. One of the scenarios related to resistance focuses on epigenetic changes. The most important of the failures observed in EOC and commonly in many neoplasms is the inactivation of *TP53* gen [4]. On the other hand, drug resistance can be caused by the treatment itself.

The genotoxic effects caused by chemo- and radiotherapy are the same ones that promote the onset and maintenance of cancer [5]. However, the consequences of these conventional treatments do not only concern genetics but also epigenetics. Tumor cells treated with chemotherapeutic drugs can undergo significant epigenetic changes, which in turn can lead to the tumor exhibiting resistance to treatment, mainly due to two important events—CpG island methylation and histone acetylation—mechanisms that can be intentionally modulated by epigenetic drugs [6]. Furthermore, it has been shown that epigenetic alterations also influence the response to cancer chemotherapy by regulating the expression of genes important in the absorption, distribution, metabolism, and excretion of chemotherapeutic drugs [7].

There is ample evidence of the association between the methylation of certain genes and conventional drug treatments for cancer. One example of this is the role played by the *RASSF1A* gene, as its methylation has been shown to be directly related to treatment response in several types of cancer. For example, it has been observed that hypomethylation of this gene in breast cancer patients treated with tamoxifen is associated with a positive response, while, conversely, reactivation of methylation implies resistance [8].

Another typical example of this interaction focuses on the *MGMT* gene, which encodes a protein that repairs DNA damage caused by alkylating agents used in cancer treatment. The relationship between methylation of this gene and cancer prognosis has been interestingly documented in glioblastomas. It has been identified that patients who exhibit methylation of this gene, and therefore whose tumor cells do not express this repair protein, benefit from temozolomide therapy [9]. In fact, it has also been shown that in patients with grade II glioma, methylation of this gene is a significant prognostic biomarker in combination therapy with radiotherapy and temozolomide [10]. Regarding EOC, a representative case in this context is evident in the role of *BRCA1*. The activation of this gene due to mutations is associated with an increased risk of developing ovarian and breast cancer, as *BRCA1* is involved in genomic stability. However, it has been identified that *BRCA1* methylation can be a useful tool for evaluating the effectiveness of treatment in these types of patients. Therefore, *BRCA1* promoter methylation is a biomarker of better response to platinum–taxane-based therapy in sporadic epithelial ovarian cancer [11] and with regard to drugs that act as PARP inhibitors [12]. Continuing with EOC, it has also been observed that methylation of the *PAX9* gene and the consequent inhibition of its expression could benefit patients with this type of neoplasm treated conventionally with platinum salts [13].

Based on the above, it is disconcerting and ironic that genotoxic agents are currently preferred in cancer treatment over other substances that can act more specifically on the real targets without causing damage to normal cells and are also more effective. Selective cytotoxicity is a characteristic of anti-tumor biocomposites obtained from plants. An example of this is compounds derived from fruits, such as *Annona muricata* (soursop) extract, which is 10,000 times more effective and highly selective in destroying HT-29 colon tumor cells than doxorubicin [14], which has profound side effects.

For more than five decades, natural products have been positioning themselves as weapons in the battle against cancer. A significant contribution from nature comes from plant alkaloids, taxoids, and podophyllotoxins [15,16,17,18,19].

The therapeutic advantages of natural compounds, particularly their lower toxicity profiles and their capacity to simultaneously modulate multiple signaling pathways involved in carcinogenesis, may partly account for the observation that, of the 240 anticancer agents approved over the past four decades, only 29 are exclusively synthetic. In fact, the evidence associated with the anti-tumor potential of natural products is so significant that several anticancer drugs have been approved that are fully synthetic yet incorporate pharmacophores of natural origin, specifically designed to emulate the biological activity of their parent natural compounds [20]. One of the most well-known cases in the search for anti-tumor agents in nature involves taxanes, which have a long history of use in folk medicine for breast and ovarian cancer [21]. The best-known representatives in clinical oncology are paclitaxel and docetaxel (a semi-synthetic analog of taxol), whose anticancer effects are based on promoting the polymerization of tubulin heterodimers, which leads to a halt in the mitotic cycle. Another group of well-known secondary metabolites is alkaloids. Among these, vincristine and vinblastine were the earliest plant-derived agents introduced into clinical oncology. These compounds exhibit potent antiproliferative effects through microtubule destabilization, which induces metaphase cell cycle arrest and subsequently triggers apoptotic cell death [22]. Etoposide and teniposide are semi-synthetic derivatives of podophyllotoxin and have also marked a milestone in oncology medicine. Podophyllotoxin is the main bioactive compound of *Podophyllum peltatum* L., and it has historically been used in the treatment of skin lesions. These derivatives inhibit DNA synthesis and stabilize the topoisomerase II–DNA cleavable complex, leading to cell cycle arrest at the late S–G2 phase [21].

Natural combinatorial chemistry is much more sophisticated than that developed in a laboratory because it leads to the production of exotic structures rich in functional groups [23], resulting in an incredible versatility of natural biomolecules. Around 1 million natural products are known, with more than half of them coming from plants; these are the most important anticancer products, since three-quarters of the compounds used in medicine are natural derivatives [24].

The purpose of this review is to highlight the active role that natural products can play in the treatment of EOC in an in vitro and in vivo context, highlighting the main events that they can trigger at the epigenetic level and which are the most important phytochemicals, with the aim of demonstrating nature’s potential to tackle this type of disease from the safety and efficacy of this type of approach.

## 2. Epigenetics of Epithelial Ovarian Cancer

**Figure 1:**
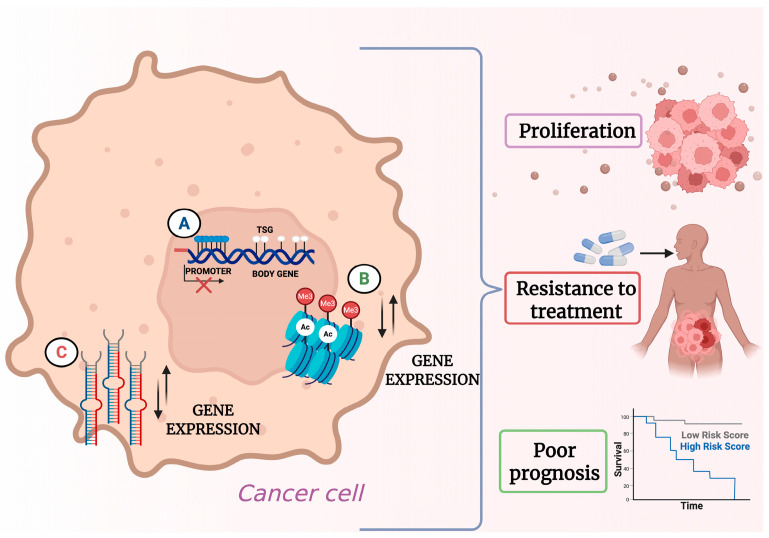
Epigenetic mechanisms of cancer and their impact on tumor biology. Various epigenetic mechanisms contribute to genetic dysregulation in cancer, promoting excessive proliferation, resistance to treatment, and a poorer prognosis. These mechanisms include the following: (A) aberrant methylation of tumor suppressor genes, which usually represses their expression when it occurs in promoter regions, accompanied by global hypomethylation; (B) biochemical modifications of histones, such as methylation (Me3) and acetylation (Ac), which regulate chromatin structure and gene expression; (C) post-transcriptional regulation mediated by microRNAs, which modulate the expression of tumor suppressor genes and oncogenes.

Regarding the epigenetic mechanisms that mediate platinum resistance in EOC, Sharma et al. postulated in 2010 that epigenetic alterations, in this case through chromatin modification, are related to the strategies used by tumor cells to evade the action of this type of drug [25]. Although the field of epigenetics includes both the study of chromatin and the role of non-coding RNA, the assessment of DNA methylation status is the most widely used strategy for understanding resistance in the epigenetic context (Figure 1).

### 2.1. DNA Methylation

A large number of studies support the relationship between methylation in CpG islands and the onset or course of cancer, as well as response to treatment. In malignant cells, global hypomethylation is identified, as well as specific hypermethylation (Figure 1(A)). The former leads to microsatellite instability, failure of transposon activation, DNA/chromosome rearrangements, and alterations in chromosome conformation [26]. The latter scenario, i.e., locus-specific hypermethylation, usually occurs in CpG islands coinciding with promoter regions. Hypermethylation is often associated with the silencing of TSGs related to DNA repair, growth control, and cell proliferation, among other mechanisms. On the other hand, hypomethylation is usually synonymous with the activation of expression, and in cancer, it involves genes related to oncogenesis [6].

In the case of EOC, there is ample evidence regarding the relationship between tumor suppressor gene (TSG) methylation and poor prognosis for this disease. Some of these studies are described in Table 1.

Within acquired resistance, an important role has been attributed to the methylation of the pro-apoptotic *hMLH1* gene, one of the most studied in ovarian cancer. Patients with EOC show an increase in the methylation of this gene when the disease recurs and after four or more cycles of platinum-based chemotherapy [27]. A similar situation occurs for genes such as *RASSF1A*, *HOX10*, and *HOX11*, whose treatment with methylation inhibitors favors the response to carboplatin in recurrent patients, with a positive correlation observed between the restoration of the activity of these genes and prolonged disease-free periods [28].

Methylation of the *BRCA1* gene promoter has been extensively analyzed in both ovarian and breast cancer. Such hypermethylation leads to the silencing of this gene [29] and is found in 5 to 22% of sporadic cases of EOC. This event is frequently associated with loss of heterozygosity at this locus and with a poor prognosis in these patients. It has been demonstrated in breast and ovarian tumor lines that *BRCA1* methylation is related to sensitivity to PARP (poly adenosine diphosphate-ribose polymerase) inhibitor drugs [30], a situation that may become a future treatment alternative in patients with these types of cancer.

**Table 1:** Methylation in EOC.

Gen	Percentage of Methylation	Analyzed Samples	References
High methylation			
*OPCML*	33–83%	Serous and non-serous	[31,32,33]
*HSulf-1*	75%	Not specified	[34]
*GATA4*	60%	Clear cells, endometroid	[35]
*CDH13*	13–67%	Clear cells, endometroid, mucinous, undifferentiated, adenocarcinoma	[36,37]
Intermediate methylation			
*DAPK*	0–67%	Clear cells, endometroid, mucinous, undifferentiated	[38,39]
*BRCA1*	5–31%	Seroso, clear cells, endometroid, mucinous, undifferentiated, adenocarcinoma	[39,40]
*CDKN2A (p16*)	0–41%	Serous, clear cells, endometroid, mucinous, undifferentiated.	[41,42]
*HOXA9*	51%	Serous, clear cells, endometroid, mucinous	[37]
*RASSF1A*	10–50%	Serous, clear cells, endometroid, mucinous, undifferentiated, adenocarcinoma	[37,39,43,44,45]
*IGFBP3*	44%	Serous, clear cells, endometroid, mucinous, undifferentiated	[46]
*APC*	11–47%	Serous, clear cells, endometroid, mucinous, undifferentiated, adenocarcinoma	[39,47]
Low methylation			
*FANCF*	0–28%	Serous and non-serous	[48,49]
*MLH1*	2–13%	Serous, clear cells, endometroid, mucinous, undifferentiated	[41,44,50]
*HOXB5*	12%	Serous, clear cells, endometroid, mucinous	[37]
*CDKN2B*	0–19%	Serous, clear cells, endometroid, mucinous	[50,51]
*PTEN*	8–17%	Serous, clear cells, endometroid, mucinous	[42,44,45]
*MGMT*	4–9%	Clear cells, endometroid, mucinous, undifferentiated	[44,47]

This table describes the main genes found to be methylated in the different subtypes of EOC, grouped according to their methylation frequency.

Among the most frequently methylated genes in EOC is *OPCML*, which encodes an adhesion molecule and whose overexpression has been shown to decrease cell growth both in vivo and in vitro in ovarian carcinoma [33,52]. Different studies have evaluated the methylation of this gene. Xing et al. determined a methylation frequency of 77% in tumors, as well as an association of methylation with tumor stage and subtype, finding it more frequently methylated in mucinous and serous tumors [31]. Another gene with a high methylation frequency is *DAPK*. Collins et al. described the methylation of this gene in 20 of 30 patients (67%) [38]. *DAPK* is a pro-apoptotic kinase that plays a role in the development of metastasis and whose methylation is manifested in various cancers [53].

Within the moderate hypermethylation levels, *HOXA9* is hypermethylated in 51% of cases, with a correlation between methylation, tumor stage, and subtype, with higher methylation in early-stage tumors and in the endometrioid type [37]. *GATA4* is also found in this group, with hypermethylation observed in 30% of endometrioid samples [35]. *GATA4* belongs to a family of transcription factors involved in morphogenesis and cell differentiation. *RASSF1A* is a TSG whose expression inhibition has been linked to methylation, with a frequency of between 10% and 50% in ovarian tumors. Zuberi et al. found this gene methylated at a frequency of 34% when analyzing all subtypes, finding a significant relationship between the serous and mucinous subtypes [45]. An important feature of this gene is that its pharmacological reactivation mediates resensitization in patients treated with platinum, with an increase in progression-free periods [28].

Another important TSG is *PTEN*, whose role has been highlighted in numerous tumors. Its lipid phosphatase activity is critical in its anti-oncogenic function as it antagonizes the PI3K-AKT/PKB pathway. Fortunately, this gene has a low methylation frequency in EOC, with methylation found in 8–17% of the different subtypes [45]. Like *PTEN*, *hMLH1* belongs to the group of genes that have a low methylation frequency, between 2 and 13% in EOC [50,54,55]. The function of *hMLH1* is to recognize DNA damage and activate apoptotic signals. Inhibition of *hMLH1* expression has been observed in relapsed patients [56]. Similar to *RASSF1A*, decreasing *hMLH1* methylation through drugs allows for gene re-expression [57]. 

### 2.2. Chromatin Modifications

Understanding the role of epigenetic marks that regulate chromatin status, such as histone acetylation and other post-translational modifications, is key to understanding the events that lead to excessive proliferation and drug resistance. Biochemical marks on histones, including acetylation, are being explored as molecular markers that define oncogenic potential in different tumors, along with the prediction of clinical outcomes [58]. Acetylation is associated with the activation of gene expression, as it promotes a decrease in the positive charge of histones, leading to an open chromatin state, while methylation does not alter the charge but influences transcription positively or negatively, depending on the residues that are methylated.

It has been identified that the activity of chromatin-modifying enzymes may be altered in EOC. For example, there is a relationship between elevated levels of H3K9 methyltransferase G9a, which promotes chromatin compaction and thus the silencing of TSGs such as *DSC3*, *MASPIN*, and *CDH1* [59,60] in different subtypes of serous ovarian cancer, a fact that has an impact on lower survival rates in these patients, since this overactivity has been associated with metastatic events [61].

This same enzyme, together with another histone methyltransferase known as GLP or EHMT1, has been shown to have a direct involvement in resistance to PARP inhibitors, which are proteins that mediate DNA repair and whose inhibitors have been positioned as a therapeutic alternative in some cases of EOC. Watson et al. (2019) observed that in cells derived from high-grade serous tumors resistant to PARP inhibitors, there is high activity of G9a and GLP and that deliberate disruption of this enzymatic activity sensitizes the cells to treatment [62].

Another event associated with the change in chromatin status is histone deacetylation, which leads to reduced transcription. It has been observed in EOC that inhibition of a histone deacetylase (HDAC6) in tumors with ARID1A mutations promotes p53 acetylation. ARID1A is a subunit of the SWI/SNF complex (chromatin remodeling machinery) that is mutated in more than 50% of EOC cases. Acetylation of p53 restores apoptotic and tumor suppressor functions, as evidenced by improved survival in mice with ARID1A mutations treated with ACY1215, a small molecule that inhibits HDAC6, which in turn deacetylates P53 [63].

As the role of biochemical modifications of histones in the genesis of ovarian cancer, as well as in its progression and prognosis, is further explored, in vitro and in vivo therapeutic approaches are being tested, in which epigenetic drugs such as deacetylase inhibitors and histone methyltransferase inhibitors are being evaluated, with positive effects observed in terms of sensitizing cells and patients to chemotherapeutic treatments.

### 2.3. Non-Coding RNA

For several decades, non-coding RNA has been gaining importance in understanding the pathophysiological behaviors of various diseases, including cancer. Non-coding RNA molecules refer to those RNA sequences that do not code for any protein (about 98.5% of the human genome). There is a wide variety of these molecules, which are grouped according to their size and regulate different processes. Long non-coding RNA (IncRNAs) and microRNAs are critical regulators in the development and progression of ovarian cancer. These molecules have been observed to play a crucial role in malignant cellular processes, such as proliferation, apoptosis, invasion, and metastasis, primarily because they are dysregulated and, therefore, affect the molecular processes that they control.

IncRNAs are RNA transcripts of more than 200 nucleotides that exhibit various functions, including transcriptional and post-transcriptional regulation, and some are involved in the pathogenesis of ovarian cancer. These molecules can act as oncogenes or TSGs. An example of this type of RNA and its involvement in ovarian cancer is HOTAIR. This type of lncRNA is overexpressed in ovarian cancer, and it has been shown that deliberately blocking its expression in vitro increases sensitivity to cisplatin in modified ovarian cancer cells [64]. HOTAIR is an lncRNA transcribed from the HOXC locus. HOX genes are a group of genes associated with embryonic development and cell differentiation processes in adult stages, and a large number of lncRNAs have been found to be transcribed from these gene groups. HOTAIR levels are positively associated with OEC staging, tumor histological grade, lymph node metastasis, and disease prognosis [65,66].

On the other side of the spectrum are tumor suppressor lncRNAs, which can inhibit cancer progression by modulating gene expression or acting as endogenous RNA competitors that sequester miRNAs. This is the case of MEG3, an lncRNA that has been downregulated in EOC and is associated with the inhibition of tumorigenesis and the progression of several types of cancer, including ovarian cancer [67,68,69]. The low expression of this molecule appears to be due to methylation of its promoter region, an event that has been observed in both EOC biopsies and cell lines. It has been observed that the induction of MEG3 expression in ASC tumor lines leads to the inhibition of proliferation through the induction of apoptosis [68].

Another type of non-coding RNA is the well-known miRNAs, whose size varies between 20 and 25 nucleotides. Their role in cancer has been of great relevance since these molecules have been studied for their role as possible biomarkers for early diagnosis and prognosis, as well as potential drug targets. miRNAs act mainly by binding to target mRNAs, causing their degradation or translational repression; therefore, their role in epigenetics lies in post-transcriptional regulation.

In the pathogenesis of EOC, deregulation of a large number of miRNAs has been observed, including miR-29b, miR-125 b, miR-29a, and let-7, which are exceptionally expressed in normal ovarian tissue. Thirty-nine miRNAs in tumor tissue are significantly inhibited compared to normal ovarian tissue. On the other hand, miRNAs are overexpressed in EOC, such as miR-141, miR-200a, miR-200c, and miR-200b [70].

## 3. Epithelial Ovarian Cancer Therapy

EOC accounts for approximately 90% of malignant ovarian tumors and continues to be the leading cause of death from gynecological cancer worldwide. This disease is notoriously silent in its early stages, contributing to late diagnosis, with more than 70% of cases identified in stages III or IV, when the disease has already metastasized diffusely to the peritoneum [71].

The genetic, molecular, epigenetic, and therapeutic response diversity exhibited by this disease, coupled with the frequent emergence of resistance to platinum-based chemotherapy, has driven the development of individualized therapeutic approaches that attempt to integrate clinical, molecular, and evolutionary information to optimize response and reduce relapse rates [72].

Over the past two decades, cytoreductive surgery and carboplatin- and paclitaxel-based chemotherapy have remained the gold standard of treatment [73]. Limitations in terms of toxicity, heterogeneity of response, and recurrence have driven the search for complementary strategies. In this context, innovative approaches have emerged, such as biomarker-based targeted therapies, evolutionary medicine applied to cancer, radiogenomics, and biomedical engineering, which seek to improve therapeutic efficacy and reduce adverse effects in the treatment of ovarian cancer [71,74,75]. These new therapeutic modalities offer substantial promise, but they also present regulatory, economic, and clinical challenges that require critical evaluation for their effective integration into medical practice.

Despite these advances, EOC continues to represent a clinical challenge, which is why complementary strategies are being explored to address current limitations.

### 3.1. Conventional Therapy

Conventional treatment for this neoplasm is based on optimal cytoreductive surgery, which aims to completely resect all visible macroscopic disease. The amount of residual tumor after surgery is the most important prognostic factor for overall and progression-free survival, with complete cytoreduction being the ideal therapeutic goal for improving clinical outcomes [76]. However, in patients with advanced disease or high surgical risk, neoadjuvant chemotherapy followed by interval surgery (NAC + IS) has emerged as an alternative strategy. According to a recent systematic meta-analysis, this therapeutic modality is valid because it initially reduces the tumor burden through chemotherapy, facilitating more effective and less aggressive subsequent surgery [77].

Conventional therapy includes surgery followed by platinum-based chemotherapy, the success of which depends largely on timely postoperative recovery so that adjuvant treatment can be started without delay. In this regard, Enhanced Recovery After Surgery (ERAS) protocols have proven to be an effective tool for optimizing patient recovery [78], as they integrate a set of perioperative interventions designed to minimize surgical stress, improve pain control, promote early mobilization, and optimize nutrition, which together reduce hospital stays and decrease postoperative complications. In patients with advanced EOC, the implementation of ERAS has shown favorable results, including a significant reduction in the length of hospitalization and the rate of readmissions, without compromising safety or increasing mortality [78].

After surgery, systemic chemotherapy based on platinum compounds, mainly carboplatin, combined with taxanes such as paclitaxel, is the standard adjuvant treatment. This combination has been shown to significantly improve objective response rates, progression-free survival, and overall survival compared to previous regimens. Chemotherapy can be administered in both the adjuvant and neoadjuvant settings, depending on tumor extent and the patient’s general condition [76].

In patients with advanced disease or clinical conditions that contraindicate primary surgery, neoadjuvant chemotherapy followed by interval surgery has established itself as an effective strategy. This modality is not inferior in terms of overall and progression-free survival compared to primary surgery followed by chemotherapy and is associated with a significant reduction in surgical morbidity and better tolerance.

### 3.2. Alternative Therapies

Although cytoreductive surgery and platinum-based chemotherapy remain fundamental pillars in the treatment of EOC, their effectiveness is limited by relapses in up to 70% of advanced cases [79]. To address this, current research includes a variety of alternatives, including the following: phytochemicals that reactivate apoptotic pathways, antioxidants that modulate oxidative stress, immunotherapies that reprogram the tumor microenvironment, nanoparticles that optimize drug delivery, natural alternatives that resensitize tumor cells, therapeutic combinations that enhance synergistic effects, targeted delivery systems that reduce systemic toxicity, and dynamic monitoring that personalizes treatments.

#### 3.2.1. Immunotherapy

This alternative has been established as an innovative therapeutic strategy for ovarian cancer, combining molecular precision and the potential to overcome therapeutic resistance. From adoptive therapies with modified cells to strategies that reprogram the tumor microenvironment, this strategy offers new hope for patients with advanced disease. CAR-NK and CAR-T cells, designed to recognize specific antigens such as claudin-6 or mesothelin, have demonstrated efficacy in in vitro and in vivo models. NK-92MI cells modified with anti-claudin-6 CAR receptors induce perforin-dependent cytotoxicity and IFN-γ release, reducing tumor burden in xenotransplanted mice [80]. A complementary approach uses CAR T cells targeting mesothelin, whose efficacy has been validated in murine models, where complete tumor elimination was observed in several cases, with lasting effects for more than three months [80].

The study of TCRs and BiTE (Bispecific Antibody Engagers) antibodies also stands out as a promising tool. Preferentially Expressed Antigen in Melanoma (PRAME) is a tumor-associated antigen belonging to the cancer–testis antigen (CTA) family. This protein is expressed in several malignant and metastatic tumors, with little or no expression in normal tissues, except in the testes, ovaries, placenta, adrenal glands, and endometrium. CCCTC-Binding Factor-Like (CTCFL) is another CTA antigen that plays a role in the epigenetic regulation and metabolic reprogramming of cancer stem cells, making it a potential target for immunological therapies. TCRs reactive against PRAME and CTCFL have shown specific cytolytic activity in ovarian cancer lines, with the release of granzyme B and caspase-3 [81]. Another innovative approach uses adenoviruses with sequences encoding anti-MUC1 and IL-2 BiTE regions, which allows for the reactivation of exhausted T cells in ascites fluid. This system, by linking T cells and antigen-presenting cells, restores immune function through IL-2 production and CD8+ lymphocyte expansion, overcoming the immunosuppressive environment of ascites [82].

Although these strategies are still in preclinical or early trial phases, their integration with conventional therapies such as chemotherapy or PARP inhibitors could optimize results. For example, the combination of CAR-NK T cells with checkpoint inhibitors (such as pembrolizumab) has overcome the immune resistance of the tumor microenvironment, a critical obstacle in solid cancers [80].

#### 3.2.2. Metabolotherapy

Modulation of lipid metabolism and the tumor microenvironment also plays a key role in EOC. In this regard, transgelin-2 (TAGLN2), a protein that regulates lipid metabolism in T cells, has been studied and identified as a key mechanism of immunosuppression in EOC. In in vitro studies, overexpression of TAGLN2 in T cells reduced their ability to take up lipids, limiting their anti-tumor function. However, inhibition of this protein by RNA interference restored T-cell activity, suggesting a pathway for enhancing immunological therapies [83].

Metabolic modulation also plays a key role in chemotherapy resistance in EOC. Inhibition of palmitoyltransferase ZDHHC12, an enzyme that mediates post-translational modification of lipids, sensitizes tumor cells to cisplatin through mechanisms mediated by reactive oxygen species (ROS), which induce apoptosis, improving response to treatment [84]. On the other hand, blocking SF3B1, a component of the splicing complex, favors the immune microenvironment by inducing pyroptosis. This inhibition enhanced the efficacy of the anti-PD-L1 antibody (αPDL1), thus demonstrating a synergy evidenced in preclinical and in vitro models [85].

#### 3.2.3. Nanoparticles

Nanoparticles and targeted drug delivery represent a revolutionary advance in the treatment of EOC, enabling precise delivery of therapies while minimizing systemic toxicity. These strategies leverage unique properties of nanoscale materials to overcome resistance and improve anti-tumor efficacy. Mesoporous silica nanoparticles (MSNs) loaded with interfering RNA (siRNA) have demonstrated the ability to deactivate the TWIST oncogene in murine models of ovarian cancer, reducing chemotherapy resistance and tumor growth [86].

Antibody-conjugated nanoparticles have shown promising selectivity toward tumor cells by recognizing specific membrane proteins. Recent research has developed a magnetic system based on iron oxide nanoparticles functionalized with antibodies targeting ovarian cancer cells. This system, loaded with therapeutic agents such as Ca (OH)_2_ and Taxotere, showed a 78.3% reduction in tumor growth in cell models, ex vivo tissues, and mice, thanks to the controlled release of drugs through photothermal activation [87].

Other approaches employ innovative hybrid systems that expand therapeutic possibilities. For example, exosomes–liposomes loaded with tryptolide (TP) and miR497 have been shown to overcome chemoresistance in ovarian tumors by modulating autophagy and the PI3K/AKT pathway, achieving 75% tumor inhibition in in vivo models [88]. The study of other natural melanin nanoparticles has demonstrated photothermal/photodynamic activity, which induces the death of ovarian cancer cells through the generation of reactive oxygen species (ROS) and localized hyperthermia [88].

#### 3.2.4. Epigenetic Approach

Epigenetic modifications have been another area of focus for the development of conventional drugs that aim to modify biochemical changes such as DNA methylation and histone acetylation, i.e., changes related to events such as oncogenesis and resistance to platinum salts observed in ovarian cancer. The clinical application of epigenetic drugs like DNA methyltransferase inhibitors (DNMTis) and histone deacetylase inhibitors (HDACis) emerged in the early 2000s, initially focusing on hematological malignancies. The DNMT inhibitors 5-azacytidine (AZA) and 5-aza-2′-deoxycytidine (decitabine) received FDA approval in 2004 and 2006, respectively, for the treatment of myelodysplastic syndromes, whereas the HDAC inhibitor suberoylanilide hydroxamic acid (SAHA) was approved in 2006 for patients with persistent or cutaneous T-cell lymphoma [89].

In ovarian cancer, the biological effects of demethylating agents have been assessed using cells derived from ascitic fluid, revealing a clear gene-specific decrease in DNA methylation levels [28]. In ovarian cancer cell lines, treatment with DNMTi has been shown to enhance the expression of key antigen-processing and presentation molecules, including B2M, CALR, CD58, PSMB8, and PSMB9, suggesting a potential mechanism by which epigenetic modulation may increase tumor sensitivity to immunotherapeutic approaches [90]. It has been shown that in high-grade epithelial carcinoma, the loss of 5-hydroxymethylcytosine (5-hmC) is a characteristic epigenetic feature associated with a low overall survival rate, shorter times to relapse, and poor response to platinum-based chemotherapy [91]. Retreatment with DNMTi restores the loss of 5-HMC and sensitivity to platinum-based chemotherapy. It has also been shown that the DNMTi guadecitabine, in combination with the PARPi talazoparib, increases the sensitivity of OC cells to PARPi, regardless of *BRCA* status [92].

The use of this type of approach has also been extrapolated to clinical practice, significantly reversing resistance to platinum salts, thanks to the use of DNMTIs. It has been observed that DNA hypomethylation caused by the use of decitabine correlates with a good prognosis. Decitabine has shown superior efficacy compared with 5-azacitidine in platinum-resistant ovarian cancer patients, achieving a 35% objective response rate and a median progression-free survival of 10.2 months, which correlated with the demethylation of the tumor suppressor genes *MLH1*, *RASSF1A*, *HOXA10*, and *HOXA11* [28]. The aforementioned AZA and SAHA have been evaluated in clinical trials with platinum-resistant/refractory ovarian cancer patients. Although SAHA showed no significant anti-tumor activity, treatment with AZA resulted in partial responses but was accompanied by considerable toxicity, including fatigue and myelosuppression [93]. On the other hand, treatment with the HDACi belinostat in platinum-resistant ovarian cancer resulted in severe toxicities without clinical benefit, leading to early study termination [89]. Similarly, vorinostat combined with carboplatin or gemcitabine caused substantial hematological toxicity despite partial responses, prompting discontinuation of the trial [94].

Despite numerous efforts to demonstrate the efficacy of these synthetic epidrugs at the clinical level, most studies have failed to demonstrate significant anti-tumor efficacy when they are used as individual agents. The combination of epimodulators is supported by their mechanistic synergy, whereby DNMTi-mediated chromatin relaxation and gene reactivation are further enhanced through HDAC inhibition. Moreover, combination regimens enable dose reduction, thereby mitigating toxicity while maintaining therapeutic efficacy. There is currently a shortage of clinical trials specifically investigating DNMTi + HDACi combinations exclusively in ovarian cancer (most are preclinical or combined with other therapies); however, combination therapy remains an attractive therapeutic goal, provided selective agents with non-synergistic toxic profiles are combined.

#### 3.2.5. Natural Products (Sulforaphane and Indole-3-Carbinol)

Cruciferous vegetables, such as broccoli, cabbage, and cauliflower, are rich sources of sulforaphane and indole-3-carbinol, natural components that have been proposed as possible adjuvants to traditional chemotherapy against epithelial ovarian cancer due to their anti-tumor effects [95]. Sulforaphane has exhibited the ability to inhibit cell proliferation [96,97,98,99] and induce apoptosis in in vitro assays using epithelial ovarian cancer cell lines [99,100,101,102]. Chen et al. demonstrated that sulforaphane, combinatorially with epigallocatechin gallate, enhances cisplatin-mediated apoptosis in ovarian cancer cells through potentiating G2/M arrest and p21 upregulation [100]. Several studies have proposed sulforaphane as a synergistic candidate for traditional chemotherapy due to its ability to enhance sensitivity to cisplatin [103,104]. Indole-3-carbinol is another molecule with anti-tumor effects. It has already been evaluated as part of chemotherapy in patients with stage III–IV serous epithelial ovarian cancer, in whom it increased progression-free survival (PFS) and overall survival (OS) at 5 years. For this reason, it is proposed as a new and promising way of maintenance therapy in advanced ovarian cancer patients [105]. In vitro assays also suggest indole-3-carbinol as a good natural therapeutic candidate in combination with conventional chemotherapy. Kelly et al. demonstrated that indole-3-carbinol inhibits 3D tumor growth, promotes apoptosis, and suppresses the ability of epithelial ovarian cancer cells to migrate, invade, and form colonies. Additionally, this molecule showed immunomodulatory capacity by inhibiting the secretion of pro-tumor cytokines (IL-6, IL-10, and IL-11) and promoting the expression of metalloproteinases (MMP8, MMP10, and MMP14) in human ovarian cancer samples [106]. Indole-3-carbinol has been shown to act synergistically with bortezomib, causing cell cycle arrest, inducing apoptosis, and inhibiting tumor growth [107], and with resveratrol, causing cell detachment, death by apoptosis, and inhibition of proliferation in a dose-dependent manner [108]. These findings propose sulforaphane and indole-3-carbinol as natural anti-tumor molecules to be included in the treatment regimen against epithelial ovarian cancer; however, more clinical trials are needed to confirm their safety in the treatment of the most lethal gynecological cancer.

Other therapeutic strategies in EOC include approaches that combine epigenetic modulation with conventional and immunological therapies, targeted therapies, and natural compounds, expanding the options for overcoming resistance and improving the efficacy of existing treatments.

## 4. Mechanisms of Action of Natural Products in Cancer

Formal pharmacological research into cancer dates back more than six decades, but a large number of anticancer compounds targeting specific pathways have not yielded good results. Therefore, there is a clear need to investigate alternative products, specifically those based on nature, which have varied characteristics to achieve better results and whose potential positions them as a benchmark for investment in research.

This need has led the scientific community to gradually focus on bioprospecting, and various therapeutic products currently in use come from natural sources, including those based on alkaloids, taxanes, and flavonoids [109]. Natural products offer an alternative therapeutic approach due to their accessibility, applicability, and reduced cytotoxicity, playing an important role in cancer treatment through tumor modulation.

It is well known that the development of cancer involves a series of “negative” events associated with biological pathways related to cell proliferation, apoptosis, repair mechanisms, and metabolic regulation, among others, and it has been observed that secondary metabolites present in biological derivatives have the ability to influence these events in a “positive” way and either promote the death of the malignant cell or reverse its transformation.

### 4.1. Mechanisms Associated with Cell Survival

It has been observed that a large number of phytochemicals have the ability to promote and activate a well-studied process of cell death known as apoptosis. Flavonoids, which are a diverse group of phytonutrients comprising a family of almost 6000 members, apart from giving fruits and vegetables their organoleptic characteristics, play an important role in inducing apoptotic events in tumor cells, mainly due to their antioxidant capacity, induction of cell cycle arrest, and inhibition of proliferation [110].

**Figure 2:**
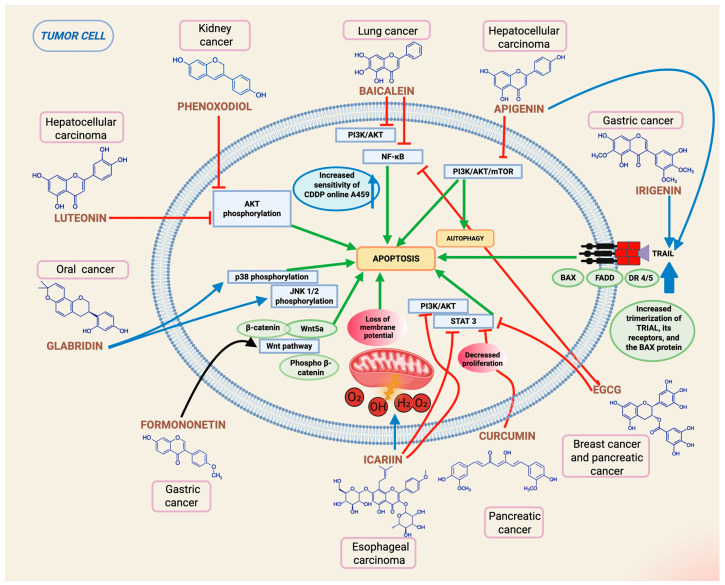
Pro-apoptotic molecular events induced by phytochemicals. Some events caused by the effect of secondary metabolites in different neoplasms are illustrated, which ultimately result in the induction of apoptosis. The red lines indicate inhibition, black arrows indicate decreased activity, blue arrows indicate increased activity, and the green ones indicate induction of the event.

Many of these molecules activate apoptotic pathways in tumor cells through their orthodox signaling pathways. For example, the isoflavone analog phenoxodiol induces apoptosis in renal cancer by inhibiting the Akt pathway (Figure 2) [111,112]. It should be noted that there are a large number of flavonoids that mediate activity through this pathway. One of them, luteolin, induces caspase-dependent apoptosis in hepatocellular carcinoma by inhibiting Akt phosphorylation (Figure 2) [113]. On the other hand, icariin glycoside induces apoptosis through ROS-mediated damage to the mitochondrial membrane potential and by suppressing the PI3K/Akt and STAT3 signaling pathways (Figure 2) [114], and baicalein, by acting on the PI3K/Akt/NFĸ-B pathway, increases the sensitivity of A549 lung adenocarcinoma cells to cisplatin, promoting apoptosis in these cells (Figure 2) [115].

Another way in which flavonoids positively influence apoptosis, which differs from the usual inhibition of Akt phosphorylation, is related to the signaling pathway involving TRAIL. Tumor necrosis factor-related apoptosis-inducing ligand (TRAIL) is a member of the TNF superfamily that selectively induces apoptosis in tumor cells by binding to and activating its death receptors DR4 and DR5. Some flavonoids can activate this pathway, such as irigenin in gastric cancer, enhancing the expression of the FAS-associated death domain protein (FADD), the expression of death receptor 5 (DR5), and the pro-apoptotic proteins Bax (Figure 2) [116]. These same pro-apoptotic events have been replicated by the action of the flavonoid pinostrobin in cervical cancer cells [117], apigenin (Figure 2) [118], and kaempferol in EOC tumor cell lines [119].

Other groups of molecules, such as polyphenols, have shown strong pro-apoptotic evidence in cancer, including curcumin. This polyphenol has been shown to block the activation of the transcription factor STAT3 at the Tyr705 residue and downregulate its expression, thereby promoting the inhibition of cell proliferation and the induction of apoptosis in pancreatic cancer cells (Figure 2), and it is also capable of inducing apoptosis in tumor stem cells and the proteolytic activation of caspases 3 and 8, as well as PARP cleavage [120]. One of the effects of another polyphenol, resveratrol, as an anti-tumor agent in gastric cancer is to promote the inhibition of the Hedgehog signaling pathway [121]. Epigallocatechin-3-gallate (EGCG), which is extracted from green tea, has shown antioxidant, anti-inflammatory, and anticancer properties in numerous experiments. EGCG has been reported to play an important role in suppressing the NFĸ-B pathway, leading to cell growth inhibition and apoptosis (Figure 2). The administration of green tea polyphenols to mice with human melanoma and to breast cancer patients sensitized tumor cells to both chemotherapy and radiotherapy by downregulating the NFĸ-B pathway [122,123]. EGCG, in combination with curcumin, can disrupt STAT3 signaling in breast cancer stem cells (Figure 2) [124]. In addition, green tea polyphenols can enhance gemcitabine activation and promote PARP cleavage and caspase 3-related apoptosis in pancreatic cancer cells, as a result of STAT3 inhibition, as mentioned in previous cases [125].

In search of therapeutic alternatives for gastric cancer, Chen et al. (2011) tested the anti-tumor capacity of formononetin, a phytochemical obtained from red clover whose potential has been demonstrated in other tumors such as breast cancer in vitro and in vivo [126], as well as glioblastomas [127]. However, the effects of formononetin in its native form are not as potent, which is why Yao’s study focused on synthesizing hybrid compounds of this element together with curcumin, finding that one of the compounds showed antiproliferative and antimigratory capacity toward the SGC7901 gastric cancer line at low doses, basically due to the involvement of the AKT/mTOR pathway, as evidenced by a decrease in the phosphorylation of this pathway in a dose-dependent manner, as well as to the Wnt/β-catenin pathway, since a decrease in the levels of β-catenin, Wnt5α, and phospho β-catenin proteins was observed in the treated cells (Figure 2) [128].

Although breast cancer numbers have improved significantly thanks to advances in screening, there are types of this malignancy that are not easy to defeat, including triple-negative breast cancer (TNBC) and those that become resistant to estrogen therapy. Chonsut et al. (2019) sought to evaluate the potential and underlying mechanism of ethoxy mansonone G (EMG) in resistant tumors, but also in cells that respond to hormone therapy [129]. EMG is a potent derivative of mansonone G (MG), the most abundant phytochemical in *Mansonia gagei*. The results of Chonsut’s work showed that EMG affected estrogen-induced cell proliferation, as well as the expression of estrogen receptor (ER) target genes in ER-positive breast cancer cells. Furthermore, a synergistic effect was observed between EMG and tamoxifen on hormone therapy-resistant cells [129].

### 4.2. Mechanisms Associated with Metabolic Impairment

Deregulation of different pathways is frequently observed in neoplasms, whether alterations in the cell cycle, apoptosis, as already described, or cellular metabolism. Altering the metabolism of tumor cells has become a promising therapeutic strategy given that many tumors undergo metabolic reprogramming that confers proliferative, survival, and immune evasion advantages. One of the most characteristic and studied alterations in cancer is the increase in aerobic glycolysis, through which malignant cells generate ATP less efficiently but more quickly [130]. Other metabolic pathways of relevance to malignant cells involve modifying lipid and folate metabolism pathways, which contribute to the maintenance of redox status [131]. Modulation by bioactive compounds such as phytochemicals represents an innovative strategy to slow tumor growth.

**Figure 3:**
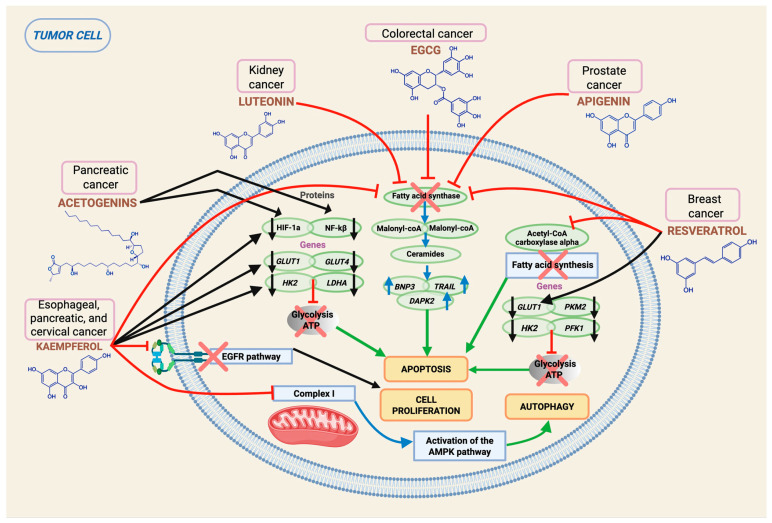
Anti-tumor metabolic changes induced by phytochemicas. Some events caused by the effect of secondary metabolites in different neoplasms are illustrated, which ultimately result in the induction of apoptosis or decrease on cell proliferation. The red lines indicate inhibition, black arrows indicate decreased activity, blue arrows indicate increased activity, and the green ones indicate induction of the event.

An interesting report on the modulatory potential of tumor metabolism focuses on the role of graviola (*Annona muricata*), a small tropical fruit tree whose components have demonstrated diverse potential for clinical applications, including cancer. The main secondary anticancer metabolites are annonaceous acetogenins, alkaloids, flavonoids, and sterols. This plant has been studied, for example, in pancreatic cancer, which is a neoplasm with a poor prognosis and resistant to conventional therapies. In a study conducted by Torres et al. (2012), the activity of graviola on the metabolism of this tumor was demonstrated, basically thanks to the negative regulation of molecules such as HIF-1a, NF-kB, and genes such as *GLUT1*, *GLUT4*, *HKII*, and *LDHA*, which are involved in glycolysis, leading to a reduction in glucose uptake by the tumor and a consequent decrease in ATP production, leading to apoptosis (Figure 3) [132]. These findings were also replicated in vivo, as there was evidence of a decrease in tumorigenicity and metastasis of orthotopically implanted pancreatic tumors [132]. The diversities in cell signal transduction caused by graviola can affect cell cycle arrest and force tumor cells to die by apoptosis or necrosis, as already described [132,133,134,135].

Flavonoids are phytochemicals well known for inducing apoptosis either by exerting direct effects on proteins such as Bax, Bcl-2, and caspases, as already described, or through the inhibition of fatty acid synthase (FAS), exerted by a large number of flavonoids, including EGCG, luteolin, quercetin, kaempferol, apigenin, and taxifolin. FAS is overexpressed in many epithelial cancers [136], and its inhibition has been shown to lead to the accumulation of malonyl -CoA, which in turn leads to the upregulation of ceramide levels and the inhibition of carnitine palmitoyltransferase-1, thereby inducing the expression of pro-apoptotic genes such as *BNP3*, *TRAIL*, and *DAPK2* (Figure 3) [137]. SAC inhibition invokes ROS overproduction, which has been described as a key factor in promoting cancer cell apoptosis.

In addition to promoting SAC inhibition, kaempferol has demonstrated anti-tumor activity by blocking glucose uptake and lactate production through the repression of genes such as *HK2* and *EGFR*. This effect has been validated in vivo and in vitro in esophageal cancer and is mediated by EGFR inhibition (Figure 3) [138]. In addition, kaempferol is capable of suppressing respiration in HeLa cells by inhibiting complex I of the mitochondrial respiratory chain, causing energy failure and inducing autophagy by increasing AMPK (Figure 3) [139].

Another compound of broad interest is resveratrol, a polyphenol derived from grapes, red wine, and red fruits, with recognized anti-tumor activity. Resveratrol acts similarly to kaempferol on glycolysis by inhibiting *HK2*, *PFK1*, *PKM2*, and *GLUT1*, and by activating AMPK while repressing mTOR, negatively modulating anabolic pathways essential for cell proliferation, for example, in breast cancer, through the consequent inhibition of acetyl-CoA carboxylase α (ACACA) and SAC [140].

A third way related to the effect on cellular metabolism is to interfere with amino acid pathways, as has been observed in the case of shikonin, a naphthoquinone compound extracted from the root of Lithospermum erythrorhizon, which promotes the negative regulation of pyrroline-5-carboxylate reductase 1 in hepatocellular carcinoma cells, an enzyme involved in proline synthesis [141].

### 4.3. Mechanisms Associated with Immunomodulation

To date, immunotherapy has achieved spectacular and lasting remission in many types of patients with advanced cancer. Tumor immunotherapy aims to restore the response in various ways, for example, by modifying immune cells, inhibiting checkpoints, and acting on the tumor microenvironment (TME), which can be targeted to overcome immune evasion by activating effector T cells [142].

**Figure 4:**
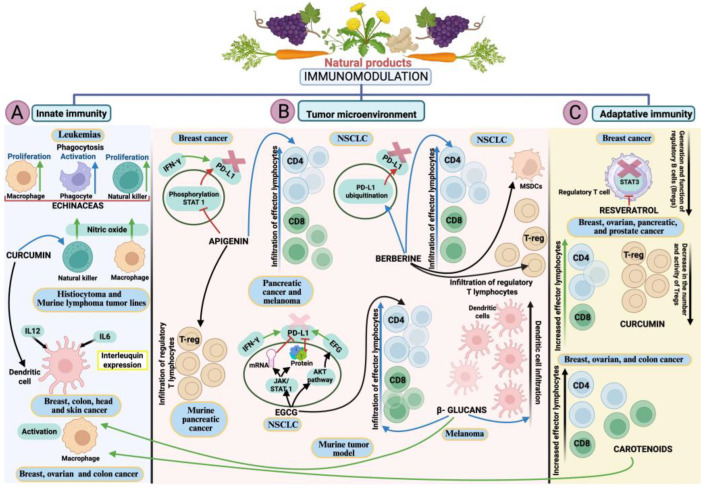
Anti-tumor immunological changes induced by phytochemicals. Various secondary metabolites have the ability to modulate events in both the innate and adaptive immune systems, as well as in the tumor microenvironment, as illustrated in the figure through different examples and in different types of cancer. The red lines indicate inhibition, black arrows indicate decreased activity, blue arrows indicate increased activity, and the green ones indicate induction of the event. NSCLC (non-small-cell lung cancer).

Several phytochemicals have demonstrated modulatory potential on immune checkpoints and the TME. For example, apigenin, a dietary flavonoid, has been shown to reduce INF-γ-induced expression of PD-L1 by inhibiting the STAT1 signaling pathway [143,144] and enhances CD4+ and CD8+ T cell infiltration in melanoma and pancreatic cancer models, promoting tumor cytotoxicity and inhibiting immunosuppression by reducing the number of regulatory T cells (Figure 4B) [144,145]. Similarly, berberine, an isoquinoline alkaloid, acts by degrading PD-L1 via the ubiquitin/proteasome pathway, simultaneously reducing the populations of immunosuppressive Tregs and myeloid-derived suppressor cells (MSDSs), thereby restoring the functionality of effector T cells in non-small-cell lung cancer (NSCLC) (Figure 4B) [146].

EGCG, a molecule mentioned in previous sections, has shown remarkable immunomodulatory effects in various types of cancer, such as lung and melanoma. Similar to other molecules, one of its main mechanisms is the control of the immune brake through the inhibition of IFN-γ-induced PD-L1 expression, both at the protein and mRNA levels in A549 lung tumor cells mediated by the downregulation of the JAK/STAT1 pathway, as well as in the Lu99 lung cell line, by controlling EGF-induced PD-L1 overexpression, but in this case through the AKt pathway (Figure 4B) [147].

Another group of compounds, not regularly mentioned but also plant-derived, are phytochemicals called polysaccharides. β-glucans, for example, have immunomodulatory capacity in tumor-associated macrophages and dendritic cells, and have been shown to significantly prolong survival prognosis in Lewis lung cancer and melanoma (Figure 4B) [148,149]. In addition, treatment with oat-derived β-glucans has been shown to alter the TME by promoting an increase in tumor-infiltrating dendritic cells, effector T cells, and memory T cells (CD4+ and CD8+ T lymphocytes), as well as activating M1 macrophages (Figure 4A) and generating pro-inflammatory cytokines such as TNF-α, IFN-γ, and IL-2 in the tumor tissues of mice bearing melanoma (B16-F10) [149].

Among the group of tumor immunomodulatory polyphenols is the well-studied resveratrol, which has been shown to regulate NK cells, T lymphocytes, and T- and B-type regulatory cells in breast cancer (Figure 4C), leukemia, and renal cell carcinoma [150,151,152]; it also promotes the infiltration of CD8+ T lymphocytes, an event that has been observed in the TEM of mice with renal cell carcinoma tumors [150]. Another polyphenol, gallic acid, has been shown to modulate tumor immunity in non-small-cell lung cancer (NSCLC) and colorectal cancer (CRC) by regulating PD-L1 expression at both the protein and mRNA levels through blocking its binding to EGFR in NSCLC cells, with the consequent suppression of PI3K and AKT phosphorylation and activation of p53 [153]. Similarly, gallic acid exerts its effects on PD-L1 protein expression in CRC cells (HT-29 and HCT 116) [154].

One of the most studied phytopolyphenols as an anti-tumor agent is curcumin, a pigment derived from the rhizomes of *Acorus calamus* L., *Curcuma longa*, and *Curcuma zedoaria*. It has been shown to regulate B cells, dendritic cells (DCs), macrophages, tumor-derived stem cells, and T lymphocytes in tumor lines in lung cancer, squamous cell carcinoma of the tongue [155,156], and breast, colon, head, and skin cancer, among others (Figure 4A) [157]. Other less studied mechanisms have been identified that also exert immunomodulatory changes by regulating ON production in NK cells and macrophages in response to murine tumor lines AK-5 (histiocytoma) and YAC-1 (lymphoma) (Figure 4A), and breast tumor exosomes [158,159]. An interesting study by Fiala et al. (2015) showed that curcumin acts synergistically with omega-3 fatty acids, enhancing NK cell-mediated apoptosis by inhibiting NFĸ-B signaling in pancreatic cancer [160]. Curcumin has also been shown to inhibit the immunosuppressive functions of Treg cells by inducing downregulation of IL-10 and TGF-β, as well as affecting macrophage and DC functions in both in vitro and in vivo models [161].

## 5. Natural Epidrugs in EOC: Epigenetic Mechanisms and Therapeutic Implications

**Figure 5:**
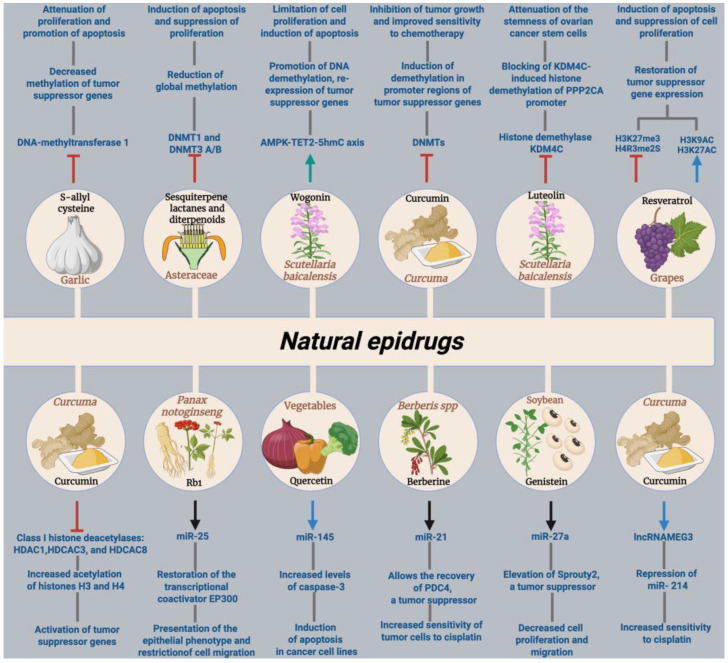
Epigenetic changes identified in ovarian cancer cell lines or tumors mediated by phytochemicals. This figure summarizes the modifications that certain phytochemicals exert on ovarian cancer cell lines or tumors at different epigenetic levels, such as changes in DNA methylation, post-translational modifications of histones, and the regulation of non-coding RNAs, and the resulting changes at the biological level. The red lines indicate inhibition, black arrows indicate downregulation, blue arrows indicate upregulation, and the green ones indicate activation.

The integration of epigenetics and phytotherapy in modern oncology represents a promising reality for the development of more effective and personalized cancer treatments. The study of natural compounds with epigenetic capacity not only expands the therapeutic arsenal against ovarian cancer but also offers alternatives with lower toxicity and the potential to overcome drug resistance, one of the main limitations of conventional chemotherapy. Evidence accumulated in recent years suggests that these compounds can precisely modulate key epigenetic mechanisms, including DNA methylation, histone modifications, and non-coding RNA regulation, thereby impacting the proliferation, migration, apoptosis, and plasticity of tumor cells. Figure 5 outlines the epigenetic effects described below.

### 5.1. DNA Methylation and Regulation by DNA Methyltransferases (DNMTs)

In the context of ovarian cancer, natural epipharmaceuticals encompass a variety of molecules that can intervene in specific epigenetic pathways. Among the most relevant mechanisms is the modulation of DNA methylation. Restoring an appropriate methylation profile is a fundamental mechanism for tumor suppression and the functional reactivation of suppressor genes. S-allyl cysteine (SAC), an organosulfur metabolite extracted from garlic, specifically inhibits the activity of DNA methyltransferase 1 (DNMT1) in A2780 ovarian cancer cells. This inhibition leads to reduced methylation in the promoter of the *CDKN1A* suppressor gene, allowing its re-expression, which attenuates cell proliferation and promotes apoptosis [162].

In a similar context, sesquiterpene lactones and diterpenoids isolated from natural sources demonstrate the ability to inhibit DNMTs (DNMT1 and DNMT3A/B), generating a significant reduction in global methylation and specific loci linked to tumor silencing in SKOV3 and OVCAR3 ovarian cancer cell lines. The functional consequence is the induction of apoptosis and suppression of proliferation, positioning these metabolites as potent epigenetic agents for complementary therapies [163].

For its part, wogonin, a natural flavonoid isolated from the roots of *Scutellaria baicalensis*, a plant used in traditional Chinese medicine, has been shown to activate the AMPK-TET2-5hmC axis in the A2780 and Kumarachi ovarian cancer cell lines, promoting DNA demethylation and the re-expression of tumor suppressor genes. Activation of this epigenetic pathway results in the limitation of cell proliferation and the induction of apoptosis in tumor cells, reinforcing the potential of flavonoids as multifunctional epigenetic agents. The ability of wogonin to modify the tumor epigenome in ovarian cancer cells highlights the importance of exploring natural compounds that act on multiple epigenetic marks, as this may translate into greater therapeutic efficacy and reduced drug resistance [164].

Curcumin, a molecule mentioned repeatedly in this study, acts directly as a DNMT inhibitor, inducing demethylation in promoters of classic suppressor genes such as *BRCA1*, which results in the re-expression of these genes in cellular models. At the same time, it represses the methylation and expression of key oncogenes such as *SNGC*, which encodes the gamma-synuclein protein, leading to marked inhibition of tumor growth and improved sensitivity to chemotherapy, demonstrating direct epigenetic modulation based on methylation [165]. In addition, curcumin affects other essential epigenetic players, such as histone deacetylases (HDACs) and histone acetyltransferases, reinforcing its multifaceted role in the comprehensive remodeling of the tumor epigenome. This specific inhibition of DNMTs by curcumin promotes increased gene expression, which leads to tumor reduction and reverses resistance to conventional drugs [166].

### 5.2. Post-Translational Modifications of Histones: Acetylation and Methylation

Covalent modifications in histones are a central therapeutic target in cancer epigenetics. Luteolin, a natural flavonoid, binds directly to histone demethylase KDM4C, inhibiting its activity. This inhibition promotes the accumulation of methylated marks on tumor suppressor gene promoters such as *PPP2CA*, leading to the repression of the PPP2CA/YAP axis and decreasing the “stemness” or ability of ovarian cancer stem cells to proliferate and resist. In this way, luteolin limits tumor progression through a specific epigenetic mechanism. This action also leads to increased sensitivity to cisplatin, increasing therapeutic efficacy [167].

Among the epigenetic modulation potential of resveratrol is a dual modulation of the histone epigenome: it decreases repressive marks such as H3K27me3 and H4R3me2s, while increasing activating marks such as H3K9ac and H3K27ac, thus restoring the expression of tumor suppressor genes such as BRCA1, p53, and p21, while also modulating microRNAs to inhibit migration and promote autophagy, thereby attenuating tumor plasticity and invasive capacity [168,169]. Another polyphenol, EGCG, exerts antineoplastic therapeutic effects through the epigenetic modulation of histone deacetylases (HDACs) and histone methyltransferases (HMTs). This dual epigenetic action allows the reactivation of tumor suppressor genes that have previously been silenced by hypermethylation or deacetylation, while inhibiting the expression of key oncogenes for tumor progression. EGCG also epigenetically regulates crucial cellular processes, such as the cell cycle, apoptosis, and DNA repair, thus contributing to the suppression of tumor growth. Although these actions have been documented in multiple types of cancer, including ovarian cancer, specific and detailed evidence on the epigenetic effects of EGCG in ovarian cells still requires further investigation for greater clinical and molecular confirmation [170].

Curcumin, with its wide range of biological effects, also acts as an epigenetic modulator, mainly through the inhibition of class I histone deacetylases (HDACs) (HDAC1, HDAC3, and HDAC8), resulting in increased acetylation of histones H3 and H4, promoting the activation of tumor suppressor genes. In addition, it modulates the activity of histone acetyltransferases (HATs) such as p300/CBP, contributing to the dynamic adjustment of acetylation and deacetylation levels. This epigenetic effect facilitates the regulation of critical cellular processes such as cell cycle arrest, apoptosis, and DNA repair. Curcumin also influences the acetylation of non-histone proteins, such as p53, increasing their tumor suppressor activity. These actions contribute to the restoration of a healthy epigenetic profile and enhance sensitivity to chemotherapeutic agents [166].

### 5.3. Regulation of Non-Coding RNA: microRNAs and Long Non-Coding RNAs (lncRNAs)

Most studies on phytochemical-mediated epigenetic modulation in cancer are based on post-transcriptional regulation controlled by non-coding RNA. The fine regulation of microRNAs and lncRNAs represents a crucial mechanism for the therapeutic action of natural epipharmaceuticals. Curcumin stands out as a key modulator of epigenetic regulation mediated by non-coding RNA in ovarian cancer. Its ability to finely influence the expression of microRNAs and long non-coding RNAs (lncRNAs) allows for the reprogramming of crucial regulatory networks that control drug resistance, apoptosis, and tumor plasticity.

Curcumin exerts one of its many epigenetic effects in ovarian cancer by demethylating the promoter of the tumor suppressor lncRNA MEG3, restoring its expression. MEG3 directly represses the microRNA miR-214, whose overexpression is associated with cisplatin resistance through the regulation of the PTEN/Akt and p53/Nanog pathways [171]. By indirectly promoting the expression of miR-214, curcumin limits the transfer of resistance mediated by extracellular vesicles, reversing chemoresistance and promoting cytotoxicity [171]. This epigenetic modulator of non-coding RNA demonstrates the therapeutic potential of curcumin to overcome drug resistance in EOC.

Curcumin has been shown to dynamically modulate specific microRNA networks in different ovarian cancer cell types, as evidenced in contrasting molecular models such as the PA1 and A2780 cell lines. In PA1 cells, this phytochemical reduces the expression of microRNAs linked to pluripotency and autophagy, notably suppressing miR-335-5p, which leads to a decrease in its molecular targets ATG5 and OCT4 [172]. Simultaneously, it promotes the expression of microRNAs with tumor-suppressing functions, such as miR-32a and miR-1285, facilitating the activation of PTEN and p53 [172]. This molecular balance creates an environment conducive to the induction of apoptosis and limits the characteristics of cancer stem cells. In addition, curcumin inhibits microRNAs associated with cell survival, such as miR-182-5p and miR-503-3p, leading to a reduction in the expression of the *BCL2* oncogene. On the other hand, in A2780 cells, this compound elevates the levels of microRNAs related to autophagy (miR-181a-3p, miR-30a-5p, and miR-216a), while repressing miR-129a-5p, a BCL2 antagonist, modulating pathways that favor cytotoxic mechanisms [172].

Curcumin has also been shown to be a chemosensitizing agent. In cisplatin-resistant ovarian cancer cells (A2780cp), the levels of miR-133b and the *GSTP-1* gene are higher than in sensitive cells (A2780). The combination of curcumin and its analog demethoxycurcumin, especially in conjunction with cisplatin, significantly reduces the expression of both miR-133b and *GSTP-1*, thereby modulating mechanisms related to drug resistance. These results suggest that curcumin acts as a natural epidrug capable of enhancing the efficacy of chemotherapy through the coordinated regulation of microRNAs (among other mechanisms) and genes involved in the response to treatment in ovarian cancer [173].

In summary, curcumin acts as a key epigenetic regulator in ovarian cancer, modulating essential microRNAs and lncRNAs that control drug resistance, apoptosis, and the tumor microenvironment. Its ability to demethylate promoters and adjust specific non-coding RNA networks enhances the efficacy of chemotherapy and opens new avenues for epigenetic therapies in this neoplasm.

On the other hand, ginsenosides 20(S)-Rg3 and Rb1 are active monomers of saponins extracted mainly from red ginseng, derived from *Panax ginseng*, and in the case of Rb1, from *Panax notoginseng*, plants widely used in traditional Chinese medicine. It has been observed that in EOC, Rb1 inhibits hypoxia-induced TEM transition by reducing the expression of microRNA miR-25, whose overexpression represses the transcriptional coactivator EP300, necessary for the expression of the epithelial marker E-cadherin. By lowering miR-25 levels, Rb1 restores EP300 and E-cadherin, preserving the epithelial phenotype and restricting cell migration linked to tumor progression [174]. For its part, 20(S)-Rg3 counteracts the Warburg effect typical of tumor metabolism by preventing lncRNA H19 from sequestering miR-324-5p, thus allowing it to repress PKM2, a key enzyme in aerobic glycolysis. This regulation significantly reduces glucose consumption and lactate production, thereby inhibiting tumorigenesis and altered metabolism in ovarian cells [175]. These results position ginsenosides derived from *Panax ginseng* and *Panax notoginseng* as epigenetic modulators that act on non-coding RNA networks to inhibit critical processes associated with invasion, aberrant metabolism, and progression of ovarian cancer.

Quercetin, a phytopharmaceutical with widely studied properties, demonstrates its therapeutic potential in EOC by modulating the regulation of microRNAs. In particular, quercetin induces apoptosis in SKOV-3 and A2780 ovarian carcinoma cells by upregulating the expression of miR-145, and this upregulation is directly associated with a significant increase in cleaved caspase-3 levels, a key marker of apoptosis [176]. These findings highlight the interaction between quercetin and microRNAs as an important mechanism in the regulation of apoptosis in ovarian cancer, positioning this natural compound as a promising therapeutic option in chemoprevention and cancer treatment [177].

Various natural compounds have been shown to play a crucial role in modulating microRNAs related to EOC progression and resistance, opening up promising avenues of research. For example, berberine has been shown to increase the sensitivity of cisplatin-resistant EOC tumor cells by reducing the expression of miR-21. This action allows the recovery of *PDCD4*, a key tumor suppressor, and in turn enhances chemotherapy-induced programmed cell death, making cancer cells more vulnerable to treatment [178]. At the same time, genistein intervenes by reducing the overexpression of the miR-27a oncogene, which results in a marked decrease in cell proliferation and migration, thanks to the elevation of Sprouty2, a regulator that slows down proliferative signals [179].

Another group of molecules with anti-tumor capacity that has already been mentioned is polysaccharides. Specifically, it has been observed that polysaccharides obtained from *Astragalus* exert a double effect: they limit the growth of cancer cells and promote apoptosis by regulating the miR-27a/FBXW7 axis (similar to what has been observed for genistein), thus restoring the suppressor role of FBXW7, a gene that plays an important role as a tumor suppressor in several types of cancer, which is blocked by miR-27a during tumor progression [180].

In a different approach, emodin, a compound belonging to the anthraquinone family, acts on (TGF)-β2 signaling through the activation of the FOXD3/miR-199a axis, inducing a significant reduction in cell viability and clonogenic capacity, demonstrating fine control over the transcriptional expression of factors involved in tumor aggressiveness [181]. Finally, icariin, one of the active compounds in the *Epidemium* plant, exerts its effects by decreasing the expression of miR-21 in EOC cell lines, which favors the restoration of key proteins for tumor inhibition, such as PTEN and RECK, while reducing the expression of Bcl-2, thus contributing to a balanced regulation between pro- and anti-apoptotic signals, efficiently promoting cell death [182].

Table 2 summarizes the main findings related to studies aimed at demonstrating the potential of natural derivatives as possible epidrugs.

This combined evidence reinforces the idea that these natural epipharmaceuticals, by reconfiguring complex networks of non-coding RNA, especially microRNAs, have the potential to change the course of ovarian cancer by directly influencing essential processes such as proliferation, migration, apoptosis, and resistance to conventional treatments. Undoubtedly, these studies open up new perspectives for the design of more specific, less toxic, and highly effective therapies against this neoplasm.

### 5.4. Pharmacology of Natural Epidrugs

Despite numerous efforts in basic science to find natural alternatives for the treatment of ovarian cancer, one of the least explored aspects in this context is the study of how these metabolites can be affected by the organism that receives them and what the consequences may be in terms of treatment effectiveness. While studies on pharmacokinetics are already scarce for natural agents in cancer in general, they are even less popular with regard to natural epidrugs in EOC.

Achieving a genuine epigenetic effect critically depends on the selection of appropriate dosing and administration schedules, as excessively high concentrations—particularly of demethylating agents—may induce cytotoxicity or conventional antiproliferative effects rather than epigenetic modulation. Moreover, when combination therapies are employed, sustained epigenetic activity during chemotherapy—and potentially beyond—is necessary to ensure effective therapeutic synergy. The pharmacological behavior of HDAC inhibitors is notably complex, as several of these agents function as enzyme inducers and can consequently alter their own pharmacokinetics upon repeated administration, as well as affect the disposition of concomitantly administered drugs. Moreover, systemic drug levels are typically low, necessitating the use of highly sensitive analytical techniques, such as high-performance liquid chromatography coupled with mass spectrometry, to ensure accurate quantification.

The pharmacokinetic (PK) profiles of epidrugs in ovarian cancer remain challenging due to limited solubility, rapid metabolic degradation, and delivery constraints, which require the development of advanced formulation approaches—such as nanoparticle-based systems or combination strategies—to optimize absorption, distribution, metabolism, and excretion (ADME). Despite these limitations, most studies on natural compounds have predominantly focused on elucidating molecular mechanisms of action rather than providing comprehensive pharmacokinetic characterization, as mentioned earlier.

Among the pharmacokinetic challenges that natural epidrugs must face and that must be studied in depth are the following: low bioavailability (poor water solubility, which limits absorption); rapid metabolism (quickly broken down by the liver, reducing effective concentration); and poor tumor penetration (difficulty reaching the tumor site effectively). The plausible strategies to consider in order to overcome these obstacles are the following: combination therapy—using natural epidrugs with traditional chemo to improve drug sensitivity and overcome resistance; delivery systems—nanoparticles or liposomes to enhance solubility, targeting, and sustained release; and structural modification—chemically altering natural compounds to improve PK properties.

Although numerous studies have described promising anticancer effects in vitro and in vivo regarding the anti-tumor potential of natural derivatives, as described in this review, and have elucidated the underlying epigenetic mechanisms, comprehensive clinical pharmacokinetic data on many natural epigenetic agents in ovarian cancer patients are virtually non-existent. Therefore, current research efforts are mainly directed toward combination-based approaches and the development of innovative delivery systems aimed at improving their therapeutic efficacy.

## 6. Preclinical/Clinical Trials of Natural Products in EOC

Preclinical trials have begun to thoroughly evaluate the efficacy of natural compounds in animal models, and more discreetly in EOC patients, with the aim of moving from basic to applied science in the context of this disease. The number of preclinical studies investigating the potential of natural products in cancer treatment has increased rapidly in recent years. These studies have provided valuable information on the mechanisms of action of natural products, their effects on cancer cells, and their ability to improve the efficacy of conventional treatments against EOC.

Resveratrol is a phytochemical that has been the subject of a large number of studies in mouse models of ovarian cancer xenografts, with the aim of evaluating the selectivity and efficacy of the molecule. In a mouse model of ovarian cancer, this phytochemical has been shown to reduce tumor progression by increasing cytotoxic T lymphocytes and antigen-presenting cells in tumor tissues. In addition, it promotes a decrease in TGF-β and an increase in IFN-γ secretion [183]. In another model, mice treated with resveratrol showed a marked decrease in glucose uptake by the tumor, which led to decreased tumor growth through the induction of apoptosis and autophagy. To obtain these findings, mice with tumors were treated with cisplatin, followed by resveratrol or the vehicle on a daily basis, and glycolysis and mitochondrial respiration in ovarian tumor cells were evaluated after treatment with resveratrol [184]. Another approach to studying the role of this molecule demonstrated the ability of resveratrol to decrease the volume and mass of PA-1 cell xenografts in athymic nude mice by inhibiting the expression of proliferating cell nuclear antigen (PCNA) and eukaryotic elongation factor 1A2 (eEF1A2) [185]. Interestingly, this study revealed the anti-angiogenic capacity of resveratrol, as evidenced by the attenuation of CD31 positivity and microvessel density in xenografts [151]. Another study reported that resveratrol decreases STAT3 expression, reduces its nuclear translocation, and positively regulates ARHI expression. Aplysia ras/ARHI homology member I (*ARHI*) is known as a TSG in EOC. These cellular effects had consequences in the inhibition of orthotopic tumor growth, assessed in terms of a significant reduction in tumor size, ascites volume, number of malignant cells, and CA125 levels [186].

Preclinical trials have also studied the effects of resveratrol combined with other compounds in EOC. For example, Fatease et al. (2019) considered testing combinations of adriamycin (ADR), which is a potent antineoplastic agent, quercetin, and resveratrol in a murine model of EOC, this group developed a polyphenol micellar system that evaluates the efficacy of ADR-sensitive (ES2-LUC) and -resistant (A2780ADR) tumor cells when ADR is administered in combination with micellar resveratrol: quercetin or micellar resveratrol: curcumin [187]. The findings showed that in the A2780ADR xenograft mouse model, only the ADR + micellar resveratrol: quercetin showed a significant reduction in tumor growth, while in the ES2-LUC xenograft mouse model, i.e., the sensitive model, all treatment regimens showed effects [187]. These results demonstrate the chemosensitizing capacity of resveratrol toward Adriamycin in this model [187].

In in vivo trials, EGCG has also been shown to inhibit the growth and metastasis of ovarian cancer cells by modulating several signaling pathways involved in tumor cell proliferation, apoptosis, and angiogenesis. For example, by studying the control exerted by PTEN as a regulator of the PI3K/Akt/mTOR signaling cascade, it has been possible to demonstrate the anti-tumor effects of EGCG treatment in vivo. The effects of EGCG on tumor cells in an ovarian xenotransplant were associated with reduced cell growth and induction of apoptosis through increased *PTEN* expression levels and a simultaneous decrease in PDK1, Akt, and mTOR expression compared to the group of mice not treated with EGCG [188]. A more recent study in 2023 also demonstrated that EGCG activates the transcription factor FOXO3A, reduces c-Myc expression, inhibits cell migration, and slows tumor growth in mouse models with SKOV 3 and A2780 xenograft A2780 [189].

It has been reported that dietary intake of flavonoids reduces the risk of cancer. In a recent preclinical study, Yang et al. (2025) analyzed the effect of the natural flavonoid wogonin in ovarian cancer models, the study demonstrated that this flavonoid, at increasing concentrations, inhibits viability and migration in human A2780 and Kuramochi cell lines; furthermore, when administered to nude mice with A2780 xenografts, the compound also reduced tumor growth without notable side effects, demonstrating both therapeutic potency and tolerability in in vivo models [164]. It was found that the effect of wogonin occurs through the activation of the AMPK pathway, which stabilizes the TET2 enzyme and increases the levels of 5-hydroxymethylcytosine (5hmC), implying a restoration of epigenetic patterns associated with tumor suppression [164].

A preclinical study used an orthotopic mouse model based on three ovarian cancer lines, SKOV3ip1, HeyA8, and HeyA8-MDR (multidrug-resistant), to test the in vivo capacity of curcumin. These mice were treated with the molecule under different regimens, and it was shown that it can inhibit growth and angiogenesis in these models by blocking NF κB, reducing IL 8,VEGF, MMP 9, and STAT3, and promoting apoptosis even in chemotherapy-resistant tumors [190]. Taking advantage of the potential of curcumin, nanoformulations of this molecule have been developed together with docetaxel (DTX-Cur/M/M nanomicelles) in order to obtain efficient release in A2780 cells. This system increased cytotoxicity, inhibited proliferation and angiogenesis, and promoted apoptosis in cells and in the animal model with a xenograft of the A2870 ovarian cancer line [191].

A little-known secondary metabolite is fangchinoline, a bisbenzylisoquinoline alkaloid obtained from an herb called *Stephania tetrandra*. This molecule has been shown to act as an inhibitor of Aurora A, a serine/threonine kinase that regulates multiple events during cell cycle progression. Its role in promoting proliferation and inhibiting cell death in cancer cells makes this kinase a target for cancer therapy. Since it is overexpressed and associated with a poor prognosis in EOC, drugs that can inhibit it have been sought. A study led by Winardi et al. (2022) revealed that fangchinoline enhances the formation of cisplatin-DNA adducts, improving the efficacy of treatment in mice with OVCAR-3 ovarian cancer [192]. By blocking the activity of a kinase critical for cell division, this compound increases the accumulation of genetic damage in resistant cells, a key finding for optimizing chemotherapy protocols [192].

Among the pioneering clinical trials that have paved the way for the therapeutic use of plant-derived natural products in EOC is one related to phenoxodiol, a genistein derivative whose use has already been described in previous sections. This molecule is a signal transduction regulator and an inducer of cell cycle arrest and apoptosis, involving multiple mechanisms, such as promoting the decrease of anti-apoptotic proteins. Phenoxodiol has been evaluated in advanced clinical trials where its role as a chemosensitizer has been revealed when combined with platinum-based drugs in patients with ovarian cancer [193]. A subsequent phase II clinical trial completed in 2011 demonstrated that the combination of phenoxodiol with paclitaxel or cisplatin was effective in patients with ovarian cancer resistant to taxanes and platinum. This study concluded that this intravenous combination was well tolerated by patients and that the cisplatin–phenoxyol combination was remarkably active [194].

Another interesting case is based on the study of combretastatins, a family of several cis-stilbenes (polyphenols) found in the *Combretum caffrum* shrub. These compounds have been shown to act indirectly on tumor cells by inhibiting tubulin polymerization, which causes disruption of tumor endothelial cells lining the tumor vasculature, inducing rapid vascular collapse in solid tumors [195]. Two types of combretastatins, A1 and A4, are naturally isolated compounds, and combretastatin A4 phosphate (CA4P), a prodrug, has been designated as an orphan drug by the FDA and approved for the treatment of ovarian cancer. In 2010, the chemosensitizing effect of CA4P was confirmed in platinum-resistant patients through a phase II trial, which showed that the addition of CA4 to paclitaxel or carboplatin was well tolerated by patients and induced a greater response than monotherapy [196].

An interesting study began in 2021 and ended in February 2025, concerning dose and safety testing of a specific product—in this case, decaffeinated coffee containing *Artemeisia annua*, an herbaceous plant primarily containing artemisinin, a sesquiterpene lactone phytochemical that has demonstrated epimodulatory potential in vitro. This trial demonstrated that ArtemiCafe^®^ Decaf, at the recommended Phase 2 dose (three cups per day), was safe and well tolerated by ovarian cancer patients who had completed their chemotherapy regimen. Key points in this study included efficacy as measured by time to tumor progression or recurrence; the ability of decaffeinated coffee to influence biomarkers of the NRF2/KEAP1 signaling pathway; and plasma concentrations of artemisinin and dihydroartemisinin. Conclusively, it was found that this product can serve as a well-tolerated alternative maintenance therapy that can potentially delay or prevent recurrence [197].

## 7. Conclusions

In vitro and in vivo research on phytochemicals as therapeutic agents in the treatment of EOC has revealed their ability to induce relevant epigenetic modifications in tumor cells. Compounds such as polyphenols, alkaloids, and terpenoids, some to a greater or lesser extent, have been shown to modulate key epigenetic processes, such as the inhibition of histone-modifying enzymes (HDACs and HATs), DNA methylation, and microRNA regulation, leading to the re-expression of GST and the inhibition of key oncogenic pathways. The research carried out reinforces the idea that natural products not only exert direct cytotoxic effects that are potentially more selective than synthetic drugs but also have the ability to remodel the tumor epigenome, thus contributing to a more stable and specific reprogramming of the malignant phenotype.

Despite the efforts and advances, the review carried out highlights specific challenges in this area. Most studies have been conducted in preclinical models, which limits the direct extrapolation of results to clinical contexts. On the other hand, the variability in the composition of plant extracts and the poor characterization of epigenetic effects (specifically with regard to DNA methylation and histone modifications) at the global and specific levels make it difficult to accurately understand the mechanisms involved. There is also a significant gap in longitudinal studies that would allow for the evaluation of induced epigenetic changes over time and their functional impact on tumor progression and therapeutic response.

Based on this exercise, it is concluded that it is critical to move toward the clinical translation of in vitro observations and preclinical models in order to obtain an accurate representation of the potential and biological effects that natural products have demonstrated in EOC. In addition, predicting the response to these compounds, designing strategies for combination with conventional therapies, and having real candidates for clinical application are essential. The study of plant molecules with epigenetic capacity represents a promising avenue for developing therapies focused on non-orthodox targets such as epigenetic events, which are also less toxic in the treatment of this disease, based on a solid molecular foundation and the exploitation of biodiversity.

## Figures and Tables

**Table 2:** Summary of the main natural epimodulators evaluated in EOC.

Compound	Source	Target	Model	Finding	Ref.
S-allyl cysteine	Garlic	Inhibition of DNA methyltransferase 1	In vitro (A2780 tumor line)	Re-expression of the *CDKN1A* TSG, attenuating cell proliferation and promoting apoptosis	[162]
Sesquiterpene lactones (dehydroleucodine, alantolactone, costunolide, and parthenolide)	Plants of the *Asteraceae* family	Inhibition of DNA methyltransferases	In vitro (SKOV3 and OVCAR3 tumor lines)	Increased expression of *MHL1* and *PTEN*. Induction of apoptosis and suppression of proliferation	[163]
Wogonin	Roots of *Scutellaria baicalensis*	Activation of the AMPK-TET2-5hmC axis	In vitro/in vivo (A2780 and Kumarachi tumor lines)	Suppression of genes associated with cell proliferation and EMT	[164]
Luteolin	Leaves and barks of celery, thyme, dandelion, clover blossoms, ragweed pollen. *Salvia tormentosa*	Binding to histone demethylase KDM4C	In vitro/in vivo (OCSLCs/Caov-3 tumor line)	Repression of the PPP2CA/YAP axis, decreasing the stemness	[167]
Resveratrol	Red grapes, berries, peanuts, and dark chocolate	Downregulation of miRNAs that target the *ARH-I* gene	In vitro (OVCAR3 tumor line)	Inhibition of migration and promoting autophagy	[169]
Curcumin	*Curcuma longa*	Demethylation of tumor suppressor lncRNA MEG3 promoter	In vitro (A2780cp tumor line)	Resensitization to cisplatin	[171]
		Modulation of cell type-specific miRNA networks	In vitro (A2780/PA1 tumor lines)	Induction of apoptosis, cell proliferation, and autophagy and decreasing of stemness	[172]
Ginsenosides (20(S)-Rg3 and Rb1)	*Panax ginseng*, *Panax notoginseng*	Rb1 reduces the expression of miR-25	In vitro (SKOV3 and 3AO tumor lines)	Restriction of cell migration linked to tumor progression	[174]
		Downregulation of the lncH19	In vitro (SKOV3 tumor line)	Reduction of glucose consumption and lactate production, inhibiting tumorigenesis	[175]
Quercetin	Fruits, vegetables, onions	Upregulation of miR-145	In vitro (SKOV3 and A2780 tumor lines)	Increased apoptosis	[176]
Berberine	Plants of the *Berberis* family	Downregulation of miR-21	In vitro (SKOV3cp tumor line)	Resensitization to cisplatin due to *PDCD4* expression restauration	[178]
Genistein	Soybeans and other legumes	Downregulation of miR-27a	In vitro (SKOV3 tumor line)	Decrease in cell proliferation and migration	[179]
Polysaccharides	*Astragalus*	Downregulation of miR-27a/FBXW7 axis	In vitro (SKOV3 and OV-90 tumor lines)	Inhibition of cell proliferation and promotion of apoptosis	[180]
Emodin	Rhubarb and aloe	Upregulation of FOXD3/miR-199a axis	In vitro (A2780 tumor line)	Reduction in cell viability and clonogenic capacity	[181]
Icariin	*Epimedium*	Downregulation of miR-21	In vitro (A2780 tumor line)	Promotion of cell death	[182]

OCSLCs (ovarian cancer stem-like cells); A2780cp (A2780 cisplatin-resistant); SKOV3cp (SKOV3 cisplatin-resistant).

## Data Availability

No new data were created or analyzed in this study. Data sharing is not applicable to this article.

## Abbreviations

The following abbreviations are used in this manuscript:

EOC	epithelial ovarian cancer
TSG	tumor suppressor gene
Tregs	regulatory T cell
MDSC	myeloid-derived suppressor cell
DNMT	DNA methyltransferase
HDAC	histone deacetylase inhibitor
INF	interferon
TME	tumor microenvironment
ERAS	Enhanced Recovery After Surgery
TP	tryptolide
ROS	reactive oxygen species
FADD	FAS-associated death domain protein
DR5	death receptor 5
EGCG	epigallocatechin-3-gallate
PARP	poly adenosine diphosphate-ribose polymerase
TNBC	triple-negative breast cancer
SAC	S-allyl cysteine
ACACA	acetyl-CoA carboxylase α
DC	dendritic cell
HMT	histone methyltransferase
ARHI	aplysia ras/ARHI homology member I
ADR	adriamycin
CA4P	combretastatin A4 phosphate
FDA	Food and Drug Administration
